# Mr. Jonathan Hutchinson’s Views on the Treatment of Senile Gangrene

**Published:** 1884-09

**Authors:** 


### Mr. Jonathan Hutchinson's Views on the Treatment of Senile Gangrene.

His paper, read before the Royal Medical and Chirurgical Society of London, began with the statement that the author's chief object was to urge the safety and expediency of amputating in senile gangrene, if the operation were done at a great distance from the disease. In the common form of gangrene of the toes and foot, the lower third of the thigh was the part suggested as the proper level of the amputation, and, in rarer cases, in which the hand was affected, the middle of the upper arm. After remarking on the fact that amputation had hitherto generally proved disappointing, owing to return of the disease, the author urged that this was owing to its having usually been done too low down. The calcification of the arteries, upon which, in the main, the disease depends, was usually greatest near the periphery, and hence the difficulty as to the supply of blood for the nutrition of the flaps. This source of danger was not met with if the amputation be done sufficiently high. In a series of cases, in very old patients, the author had not encountered the recurrence of gangrene, excepting in one. In three the stump had healed well. In a fourth, in which the patient, although not old, was prematurely senile, and the calcification of the arteries extreme, the recovery had also been excellent. In this instance the femoral artery was so rigid that it stuck out from the face of the stump like a small bone. One of the patients, in whom the stump had healed without a drawback, was seventy years old. In two of the cases the other foot had been subsequently threatened with gangrene. As to the time to be selected, the author thought that as soon as the patient was so ill as to be confined to bed and the disease was well established, it was best to operate. Spontaneous cure was, he urged, very exceptional, and a great majority of such cases ended in death, after a long period of much suffering. The thinner the patient, the less was the risk of amputation. In a few cases in which the thigh was exceptionally fat and the tissue flabby, it might be wise to hesitate as to recommending it. In all cases, Lister's precautions had been carefully used, and in two or three, the patient had never experienced the slightest pain from the day of the operation.

